# Referring Parkinson’s disease patients for deep brain stimulation: a RAND/UCLA appropriateness study

**DOI:** 10.1007/s00415-015-7942-x

**Published:** 2015-11-03

**Authors:** Elena Moro, Michael Schüpbach, Tobias Wächter, Niels Allert, Roberto Eleopra, Christopher R. Honey, Mauricio Rueda, Mya C. Schiess, Yasushi Shimo, Peter Valkovic, Alan Whone, Herman Stoevelaar

**Affiliations:** Division of Neurology, CHU of Grenoble, Joseph Fourier University, INSERM U836, Grenoble, France; Department of Neurology, Movement Disorders Center, Bern University Hospital and University of Bern, Bern, Switzerland; Assistance-Publique Hôpitaux de Paris; Centre d’Investigation Clinique 9503, Institut du Cerveau et de la Moelle épinière; Département de Neurologie, Université Pierre et Marie Curie, Paris 6 et INSERM, CHU Pitié-Salpêtrière, Paris, France; Department of Neurology and Hertie Institute for Clinical Brain Research, University of Tübingen, Tübingen, Germany; Center of Rehabilitation, Bad Gögging, Germany; Neurological Rehabilitation Center Godeshoehe, Bonn, Germany; Department of Neurology, Santa Maria della Misericordia University Hospital, Udine, Italy; Division of Neurosurgery, University of British Columbia, Vancouver, Canada; Departamento de Neurociencias, Fundación Cardiovascular de Colombia, Bucaramanga, Colombia; Department of Neurology, University of Texas Medical School at Houston, Texas, USA; Department of Neurology, Juntendo University School of Medicine, Tokyo, Japan; 2nd Department of Neurology, Comenius University Faculty of Medicine and University Hospital Bratislava, Bratislava, Slovakia; Department of Neurology, Frenchay Hospital, Bristol, UK; Centre for Decision Analysis and Support, Ismar Healthcare, Lier, Belgium

**Keywords:** Parkinson’s disease, Deep brain stimulation, RAND/UCLA appropriateness method, Referral, Consultation

## Abstract

In 2005, a European expert panel developed and validated an electronic tool to support the appropriate referral of patients with Parkinson’s disease (PD) for the consideration of deep brain stimulation (DBS). Since new evidence has become available over the last decade an update of the tool is necessary. A world-wide expert panel (71 neurologists and 11 neurosurgeons) used the RAND/UCLA Appropriateness Method to assess the appropriateness of referral for 1296 scenarios (9-point scale). Scenarios were permutations of 8 clinical variables relevant to the decision of referral. Appropriateness of referral was calculated on the basis of the median score and the extent of agreement. Compared to 2005, the impact of clinical variables on the appropriateness of referral was similar for severity of on–off fluctuations, dyskinesias and refractory tremor (positive association, *p* < 0.001), and cognitive impairment (negative association, *p* < 0.001). A relatively stronger negative impact was seen for levodopa-unresponsive gait and balance disturbances as well as older age, the latter most likely due to a higher cut-off value (75 versus 70 years in the previous study). The impact of PD duration on the appropriateness of referral was less pronounced than in 2005. The contribution of the newly included variable ‘non-motor side effects of anti-PD medication’ was very modest. Based on these results the panel produced new recommendations on the appropriateness of referral for the evaluation of DBS in PD patients. Differences from the previous study reflect the new clinical evidence, particularly related to the use of DBS in an earlier stage of PD. The validation of the updated recommendations is in progress.

## Introduction

Deep brain stimulation (DBS) is an established treatment for well-selected patients with Parkinson’s disease (PD) [1, 2]. Patient eligibility for DBS is determined by rigorous and standardised evaluation in specialised surgical movement disorder centres. However, patient pre-selection by non-specialised neurologists or other physicians is often not optimal due to lack of criteria or guidelines that are easy to use in daily practice. This may lead to inappropriate referrals [3, 4] but also to under-referrals if potentially eligible candidates are not given the opportunity to be evaluated in a specialised centre. A recent Swedish survey has revealed that three quarters of patients with advanced PD were not even informed of the possibility of having DBS therapy by their neurologists [5].

In 2005 a European expert panel used the RAND/UCLA Appropriateness Method (RUAM) [6] to develop patient-specific referral criteria for DBS consideration in patients with PD [7]. These criteria were subsequently embedded in an online decision support tool (“Stimulus”) [7]. An observational study in Germany and Spain showed that the selection rate for DBS was significantly higher for patients in which the Stimulus tool had been used compared to the unscreened population (77 versus 48 %, respectively) [8]. A recent single-centre study in the USA showed that Stimulus was superior to another tool in predicting DBS candidacy in patients with PD [9].

Over the last few years new evidence has become available on the efficacy of DBS, particularly in an earlier stage of PD [10], and with respect to non-motor fluctuations and symptoms, comprising fluctuations of mood and impulse control disorders [11]. These important data needed to be incorporated into an update of the Stimulus recommendations and the online tool. Given the world-wide application of DBS in PD, a global approach targeting at broadly applicable criteria was adopted. The aim of this study was to update the 2005 referral criteria and to develop common recommendations for DBS consideration in patients with PD.

## Methods

### Selection of panellists and construction of clinical scenarios

**Fig. 1:**
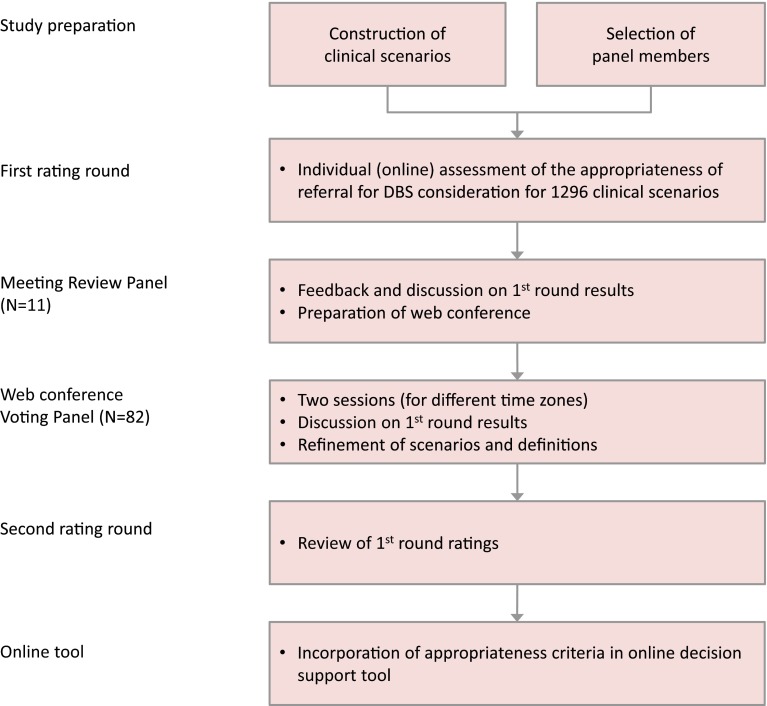
Study design

Similar to the European study [7], the RUAM [6, 12] was used to establish patient-specific referral criteria. An Executive Committee, consisting of three neurologists (EM, TW, and MS) and a methodologist (HS) prepared the study design (Fig. 1). Three of the executive committee members were previously involved in the European study [7]. Panellists were recruited from a worldwide network of DBS implanting centres. The principal selection criterion was their active involvement in the selection of at least 15 patients with PD for DBS annually. Furthermore, a reasonable geographic spread was pursued. In total, 146 neurologists and 29 neurosurgeons received an invitation by email of whom 121 (69 %) responded. Of this group, 105 physicians (87 %) agreed to participate.

Clinical scenarios were based on those of the European study [7] with some adaptations and refinements. Scenarios were permutations of the values of eight variables considered relevant to the decision of referral (Table 1). Principal changes in comparison to the 2005 study were the adapted categories for age and PD duration, and the addition of the variable ‘non-motor side effects of anti-PD medication’ (Table 1). The population considered was restricted to patients who met the absolute criteria for DBS (Table 1).

**Table 1:** Definition of terms and variables/categories used for the construction of clinical scenarios

Absolute criteria for the consideration of DBS
Parkinson’s disease for at least 4 years
Presence of bothersome disease-related symptoms (motor fluctuations, dyskinesias, persisting tremor) and/or side effects related to anti-parkinsonian medication (hyperdopaminergic, anticholinergic)
Motor improvement with dopaminergic medication or presence of medically refractory tremor
Absence of medical conditions preventing surgery (e.g., terminal cancer, severe cardio-respiratory insufficiency)
Absence of ongoing severe, medically resistant neuropsychiatric diseases (e.g., severe depression, severe cognitive impairment)
Referral
Referral for the detailed evaluation of DBS therapy in patients with PD
Irrespective of the target (subthalamic nucleus, globus pallidus pars interna, ventral intermediate nucleus of the thalamus)
Variables used for the construction of clinical scenarios
Age (<60 years; 60–74 years; ≥75 years)^a^
PD duration since onset of symptoms (4–7 years; ≥7 years)^b^
Parkinsonian signs during OFF periods, despite medical treatment (no–mild; moderate; severe impact on activities of daily living)
Dyskinesias (no–mild; moderate; severe impact on activities of daily living)
Tremor unresponsive to or requiring unacceptably high doses of anti-parkinsonian medication (no–mild; moderate; severe impact on activities of daily living)
Levodopa-unresponsive gait and balance abnormalities (no; yes)
Cognitive impairment (no–mild; moderate)
Non-motor side effects of anti-parkinsonian medication (no–mild; moderate–severe)^c^

^a^Age categories in 2005: <60 years; 60–69 years; ≥70 years

^b^PD duration categories in 2005: <5 years; ≥5 years

^c^Not included as a variable in 2005

### Panel process

The typical RUAM process consists of individual rating rounds and plenary face-to-face discussions [12]. The large size of our panel and the involvement of people from all over the world necessitated some practical adaptations, such as web conferences for the plenary meetings (Fig. 1).

Panellists used an online programme to individually rate the appropriateness of referral for 1296 different scenarios. The appropriateness scale ranged from 1 (extremely inappropriate) to 9 (extremely appropriate) with 5 being ‘uncertain’ or ‘equivocal’. Ratings had to be based on medical considerations only, and economic aspects had to be disregarded. Instructions to the ratings were accompanied by an overview of key peer-reviewed publications regarding DBS therapy for PD. After the ratings had been completed, data were analysed for patterns of appropriateness in relation to the clinical variables used.

A selection of 11 participants (Review Panel; “Appendix 1”) convened in Germany (November 2013) to discuss the results and to prepare a follow-up web conference for all panel members (January 2014). Thereafter, panellists were asked to review all their first round results and to adjust the values if thought to be necessary.

### Classification of appropriateness and statistical analysis

Classification of appropriateness was based on mathematical rules typically applied in RAND/UCLA appropriateness studies [12]. Referral was classified as appropriate if the median score was 7–9 without disagreement, and as inappropriate if the median score was 1–3 without disagreement. All other outcomes were deemed uncertain. Disagreement was defined as the situation in which at least one-third of the panellists had scored in each of the extreme sections of the 9-point scale (1–3 and 7–9) [12]. To correct for potential asymmetric ratings, the disagreement calculations were checked using the interpercentile range adjusted for symmetry (IPRAS) formula that has been advised for use in large-scale panels [12]. Logistic regression was used to determine the relationship between clinical variables and appropriateness of referral. All statistical analyses were performed using IBM SPSS for Windows version 22.

## Results

### Participants and panel process

Of the 105 physicians who agreed to participate, 82 (78 %) completed all first round ratings. This voting panel consisted of 71 neurologists and 11 neurosurgeons from 28 countries (“Appendix 1”). Ninety-four percent reported ≥5 years of experience with patient selection for DBS, and 51 % mentioned to be involved in at least 25 evaluations annually. The first online rating round was conducted in October–November 2013. Panellists needed on average 3–4 h for completing the ratings. Discussion of the results by the Review Panel and during the web conference revealed that the structure and variables were adequate, but that different perspectives had been used while doing the ratings (appropriateness of DBS versus the appropriateness of referral). Panellists were asked to review all ratings taking the perspective of appropriateness of referral as the starting point. Second round ratings were completed in April 2014.

### Agreement and appropriateness

Using the RUAM classical calculation, disagreement after the second round was found for only two out of 1296 scenarios (0.2 %). Application of the IPRAS formula resulted in disagreement for 1.2 % of scenarios. However, the two approaches never led to different appropriateness outcomes.

Referral for DBS consideration was deemed inappropriate for 15 % of the scenarios, appropriate for 46 % of the scenarios, and uncertain for the remaining 39 %. Appropriateness figures by clinical variables are given in Table 2. Logistic regression analysis (appropriate versus uncertain/inappropriate) revealed a consistent pattern of factors determining the appropriateness of referral (predictive value 96.5 % at a cut-off value of 0.5). Whereas the severity of OFF symptoms, dyskinesias and tremor showed a pronounced positive association with appropriateness, a negative impact was found for cognitive impairment, levodopa-unresponsive gait and balance problems, and higher age (Table 3).

**Table 2:** Appropriateness of referral for DBS by clinical variables

Variable	Categories	Inappropriate %	Uncertain %	Appropriate %	*p* value^a^
Age	<60 years	2	30	68	<0.001
	60–74 years	3	40	57	
	≥75 years	41	47	12	
PD duration	4–7 years	16	40	44	0.279
	≥7 years	15	38	48	
OFF symptoms	Mild	22	41	38	<0.001
	Moderate	15	41	45	
	Severe	10	36	55	
Dyskinesias	Mild	24	39	38	<0.001
	Moderate	14	43	43	
	Severe	9	35	57	
Tremor	No/mild	25	41	35	<0.001
	Moderate	15	42	43	
	Severe	7	34	59	
Gait/balance problems	No	9	26	65	<0.001
	Yes	22	52	26	
Cognitive impairment	No/mild	6	28	66	<0.001
	Moderate	25	50	26	
Non-motor side effects	No/mild	18	39	44	0.085
	Moderate-severe	13	39	48	

Percentages apply to the complete set of clinical scenarios (*N* = 1296)

Row totals per variable are 100 %, but may slightly deviate due to round-offs

^a^Pearson’s Chi-square test for categorical data

**Table 3:** Determinants of the panel outcome “appropriate” versus “uncertain/inappropriate”

Variable	Value	*β*	SE	*p* value
Age	60–74 years	−2.64	0.41	<0.001
	≥75 years	−16.06	1.42	
PD duration	≥7 years	1.27	0.32	<0.001
OFF symptoms	Moderate	1.97	0.40	<0.001
	Severe	4.89	0.55	
Dyskinesias	Moderate	1.53	0.38	<0.001
	Severe	5.47	0.59	
Refractory tremor	Moderate	2.10	0.38	<0.001
	Severe	7.25	0.72	
Levodopa-unresponsive gait/balance abnormalities	Yes	−11.34	1.02	<0.001
Non-motor side effects of anti-parkinsonian medication	Moderate–severe	1.09	0.31	<0.001
Cognitive impairment	Moderate	−11.60	1.04	<0.001
Constant value		7.04	0.85	

Outcomes of logistic regression analysis

Reference classes for regression: age: <60 years; PD duration: 4–7 years; OFF symptoms: mild; Dyskinesias: mild; Refractory tremor: no/mild; Levodopa-unresponsive gait/balance abnormalities: no; Non-motor side effects of anti-parkinsonian medication: no/mild; Cognitive impairment: no/mild

Though statistically significant, the contribution of PD duration and presence of non-motor side effects of antiparkinsonian medication was small. No meaningful interaction effects between the variables, including age and PD duration, were seen.

### Differences by subgroups of raters

Neurosurgeons showed higher rates for referral consideration than neurologists (61 versus 43 %; *p* < 0.001); similar figures were seen for the Review Panel (59 %) versus other participants (43 %; *p* < 0.001). Variations by geographic regions could not be assessed due to the small number of participants per area.

### Online educational tool

Panel results were embedded in an online tool that allows the user to select a patient profile and to see the related panel recommendation and additional information on patient selection for DBS. The tool can be freely accessed via https://www.earlystimulus.com.

## Discussion

Our study provides practical criteria to reduce inappropriate referrals and to support referral of appropriate candidates for DBS in patients with PD. This is particularly relevant since there is a strategic need for physicians and patients to improve the process quality of PD referrals for DBS.

The previously developed Stimulus tool has been shown to be able to considerably improve the quality of pre-selection by referral neurologists [8, 9]. The current study has not only updated the referral criteria taking into account new evidence from recent clinical studies [10, 11], but it has also received wide feedback for a worldwide applicability by involving expert neurologists and neurosurgeons from many countries around the world. Although some differences by subgroups were seen, this large-scale panel reached considerable agreement on the appropriateness of referral for DBS consideration in patients with PD. The relatively large area of uncertainty (39 % of indications) was not due to opposite opinions, but to “middle-of-the-road” ratings reflecting situations in which potential advantages and drawbacks of referral were considered to be equivocal. However, the appropriateness figures relate to a theoretical population, and the distribution of clinical scenarios in real-life practice may differ considerably. Moreover, some scenarios may reflect frequent constellations whereas others may be rather “constructed” and rarely seen in daily clinical practice.

**Fig. 2:**
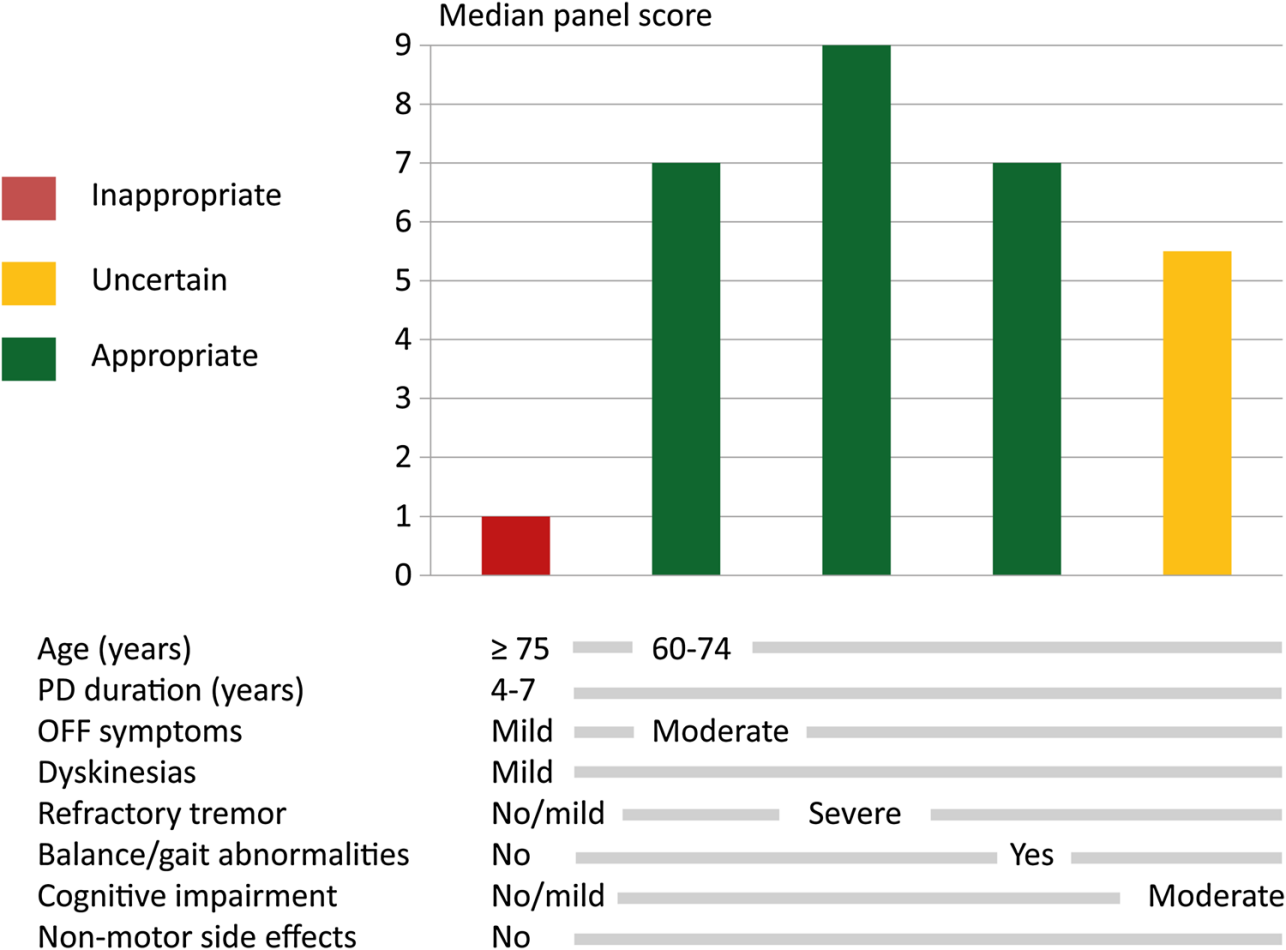
Panel outcomes for selected patient profiles

Regression analysis showed consistent and logical associations between the clinical variables and appropriateness outcomes. As no meaningful interaction effects were detected, the impact of the variables on appropriateness of referral for DBS was merely cumulative. That means that the sum of positive and negative factors, with their different weights, determines the appropriateness category. To illustrate this result, the panel outcomes (appropriateness category and median score) for five selected profiles are projected against the patient characteristics in Fig. 2.

The comparison with the results from our previous study [7] suggests similar patterns for the impact of the severity of OFF symptoms, dyskinesias and refractory tremor (positive association), as well as of cognitive impairment (negative association). In this current study, a relatively stronger negative impact was seen for older age and levodopa-unresponsive gait and balance disturbances. For older age, the difference is most likely to be ascribed to the higher cut-off value (75 versus 70 years in the previous study) as the general trend goes to including older patients.

With the new clinical evidence showing the benefits of DBS at an earlier stage of PD [10, 13], criteria of DBS selection concerning PD duration and severity have changed. This is reflected in the less pronounced impact of PD duration on the appropriateness of referral in our study in comparison to the view of the initial panel [7]. The panel ratings also indicate that these recent findings on the effect of early surgical treatment [10] are readily implemented by experts worldwide in spite of a controversial debate on this topic [14, 15].

As evidence became available that DBS may also be useful in improving non-motor side effects of anti-parkinsonian medication [11, 16], we included this condition in the construction of clinical scenarios. However, its impact on the appropriateness of referral, though statistically significant in multivariate logistic regression, was very modest. This finding could reflect the insufficient distinction made by neurologists between hyperdopaminergic behavior induced by anti-PD medications (that is supposed to improve with subthalamic nucleus (STN) DBS due to reduction of medications) and impulsivity occasionally induced by STN DBS (that may occur under inappropriate STN-DBS programming) [17].

Other factors used by the panel study, including the absolute criteria for the consideration of DBS for PD (bothersome symptoms, motor improvement with dopaminergic medication or presence of medically refractory tremor, absence of medical conditions preventing surgery, absence of severe medically resistant neuropsychiatric diseases) are comparable to the initial study [7] and have not undergone substantial changes over the last decade [18, 19].

The dissemination of the panel recommendations via a quick and user-friendly online tool has already proven its usefulness in a European setting [8]. The new updated tool, established now with the involvement of a world-wide panel, may have a larger geographic reach and impact. However, the authors’ personal experience with the first Stimulus tool has shown that users frequently misinterpreted an appropriate outcome as being synonymous to “eligible for DBS”. Therefore, we would like to emphasise that the tool is designed to assess the appropriateness of referral to a specialised DBS centre for further evaluation, and not for surgery itself.

The most important limitations of this study are related to the subjective nature of the panel opinions, and selection of panel members may, therefore, influence the outcomes. Indeed, new published guidelines or consensus about selection of patients for DBS are still lacking, despite the available evidence concerning efficacy of DBS on earlier PD stages and on non-motor fluctuations. However, various studies have shown that agreement between RUAM panels of similar composition, even if from different countries or continents, is usually satisfactory to good [20–23]. Comparison of our initial and current study into DBS/PD confirms on the one hand the reproducibility of the study results over time and across countries, but also suggests sensitivity to change in scientific insights.

In conclusion, patient eligibility for DBS is determined in specialised surgical centres following a rigorous and extensive evaluation that is often challenging for the patient and the family, both physically and emotionally. The use of clear and practical criteria for pre-selection by general neurologists or other physicians may reduce the number of inappropriate referrals, and may also help to avoid under-referral of potentially appropriate candidates. A large world-wide panel has produced detailed and consistent recommendations on the appropriateness of referral for the consideration of DBS in patients with PD. Differences in comparison to the initial panel study reflect new clinical evidence, particularly in relation to the use of DBS in an earlier stage of PD. The use of a simple online tool may help to disseminate the panel recommendations and to increase the quality of pre-selection for DBS. Research on validating the updated recommendations is in progress.

## Appendix 1

### Participants to the panel study

Niels Allert (Germany),^2^ Vladimir Alexeyevets (Belarus), Gabriel Arango (Colombia), Keyoumars Ashkan (United Kingdom), Merek Baláž (Czech Republic), Luigi Bartolomei (Italy), Oscar Bernal (Colombia), Rupam Borgohain (India), Alfonso Castro (Spain), Roberto Ceravolo (Italy), Haibo Chen (China), Ling Chen (China), Shin-Yan Chen (Taiwan), Miguel Coelho (Portugal), Arthur Cukiert (Brazil), Carsten Eggers (Germany), Roberto Eleopra (Italy),^2^ Amalia Ene (Romania), Alfonso Fasano (Canada), Tao Feng (China), Tom Foltynie (United Kingdom), Alexandre Francisco (Brazil), Milan Grofik (Slovakia), Chris Honey (Canada),^2^ Wolfgang Jost (Germany), Merete Karlsbor (Denmark), Asha Kishore (India), Peter Klivenyi (Hungary), Karina Knudsen (Germany), Norbert Kovacs (Hungary), Pierre Krystkowiak (France), Max Lange (Germany), Nilton Lara (Brazil), ChongSik Lee (Korea), Xiaoguang Luo (China), Yasushi Miyagi (Japan), Claudia Moreno, (Colombia), Francesca Morgante (Italy), Elena Moro (France),^1,2^ Mariana Moscovich (Brazil), Stefania Nassetti (Italy), Martin Nevrlý (Czech Republic), Genko Oyama (Japan), Claudio Pacchetti (Italy), Berta Pascual (Spain), Nicola Pavese (United Kingdom), Manuela Pilleri (Italy), Walter Pirker (Austria), Monika Pötter-Nerger (Germany), Carlos Rieder (Brazil), Mayela Rodriguez (Korea), Maria Jose Rosas (Portugal), Simone Rossi (Italy), Mauricio Rueda (Colombia),^2^ Hidemoto Saiki (Japan), Patrick Santens (Belgium), Mya Schiess (United States),^2^ Michael Schüpbach (Switzerland),^1,2^ Maria Chiara Sensi (Italy), Yasushi Shimo (Japan),^2^ Friederike Sixel-Döring (Germany), Martin Südmeyer (Germany), Chun-Hwei Tai (Taiwan), Atsushi Takeda (Japan), Gertrud Tamas (Hungary), Giorgio Tommasi (Italy), Wesley Thevathasan (Australia), Atsushi Umemura (Japan), Peter Valkovic (Slovakia),^2^ Franco Valzania (Italy), Tobias Wächter (Germany),^1,2^ Pettarusp Wadia (India), Jian Wang (China), Alan Whone (United Kingdom),^2^ Håkan Widner (Sweden), Tatiana Witjas (France), Karsten Witt (Germany), Qin Xiao (China) Ludvic Zrinzo (United Kingdom), and Xian-Lun Zhu (Hong Kong).

^1^Member of the Executive Committee

^2^Member of the Review Panel
